# Molecular Cloning and Characterization of a P-Glycoprotein from the Diamondback Moth, *Plutella xylostella* (Lepidoptera: Plutellidae)

**DOI:** 10.3390/ijms141122891

**Published:** 2013-11-20

**Authors:** Lixia Tian, Jiaqiang Yang, Wenjie Hou, Baoyun Xu, Wen Xie, Shaoli Wang, Youjun Zhang, Xuguo Zhou, Qingjun Wu

**Affiliations:** 1 Department of Plant Protection, Institute of Vegetables and Flowers, Chinese Academy of Agricultural Sciences, Beijing 100081, China; E-Mails: liouyun555@126.com (L.T.); jiaqiangyang@foxmail.com (J.Y.); houwenjie0628@126.com (W.H.); xubaoyun@caas.cn (B.X.); xiewen@caas.cn (W.X.); wangshaoli@caas.cn (S.W.); zhangyoujun@caas.cn (Y.Z.); 2 Department of Entomology, University of Kentucky, Lexington, KY 40546-0091, USA

**Keywords:** P-glycoprotein, cloning, characterization, *Plutella xylostella*, ABC transporter

## Abstract

Macrocyclic lactones such as abamectin and ivermectin constitute an important class of broad-spectrum insecticides. Widespread resistance to synthetic insecticides, including abamectin and ivermectin, poses a serious threat to the management of diamondback moth, *Plutella xylostella* (L.) (Lepidoptera: Plutellidae), a major pest of cruciferous plants worldwide. P-glycoprotein (Pgp), a member of the ABC transporter superfamily, plays a crucial role in the removal of amphiphilic xenobiotics, suggesting a mechanism for drug resistance in target organisms. In this study, *PxPgp1*, a putative Pgp gene from *P. xylostella*, was cloned and characterized. The open reading frame (ORF) of *PxPgp1* consists of 3774 nucleotides, which encodes a 1257-amino acid peptide. The deduced *PxPgp1* protein possesses structural characteristics of a typical Pgp, and clusters within the insect ABCB1. *PxPgp1* was expressed throughout all developmental stages, and showed the highest expression level in adult males. *PxPgp1* was highly expressed in midgut, malpighian tubules and testes. Elevated expression of *PxPgp1* was observed in *P. xylostella* strains after they were exposed to the abamectin treatment. In addition, the constitutive expressions of *PxPgp1* were significantly higher in laboratory-selected and field-collected resistant strains in comparison to their susceptible counterpart.

## 1. Introduction

The diamondback moth, *Plutella xylostella* (L.) (Lepidoptera: Plutellidae), is one of the major lepidopteran pests of cruciferous vegetables worldwide. A recent study estimated that with the intensification of agriculture, the annual crop losses and management costs caused by *P. xylostella* have increased from US$1 billion to US$4–5 billion over the past two decades [1–3]. Apart from its agricultural and economic importance, *P. xylostella* is known for its ability to develop resistance to almost all classes of insecticides, including macrocyclic lactones (MLs), potent nematicidal and insecticidal compounds derived from *Streptomyces* spp. Avermectins, an important subgroup of the MLs, consist of abamectin for pest control and ivermectin for parasite control. In the early 1990s, a 195-fold abamectin resistance was documented in *P. xylostella* field populations from Malaysia [4]. Recently, a field-derived population collected in 2007 from Yunnan Province, China, exhibited about 5000-fold resistance to abamectin and a laboratory-selected strain developed 23,670-fold abamectin resistance [5]. Abamectin resistance in laboratory-selected strains of *P. xylostella* was reported to be incompletely recessive, autosomally inherited and possibly controlled by multiple genes [6].

Although mechanisms of *P. xylostella* resistance to abamectin have not yet been fully elucidated, a number of hypotheses have been proposed. Major mechanisms include metabolic resistance, which involves the phase I, and II detoxification enzymes, including cytochrome P450 monooxygenases (P450s) and glutathione S-transferases (GSTs) [7–9]; reduced cuticle penetration of insecticides [10]; and target-site insensitivity, such as conformational changes in ligand-gated chloride channels [11–16]. Recent studies have focused on ATP-binding cassette (ABC) transporters acting in a phase III detoxification process. This can actively export conjugated toxins out of the cell and can contribute to xenobiotic resistance in insects [17–19]. Recently, 53 ABC transporters have been uncovered in the midgut of Bt resistant *P. xylostella* larvae [20], in which ABCC2, a member of ABC transporter, has been implicated in Bt resistance [21].

ABC transporters constitute a large protein superfamily and exist in all organisms from prokaryotes to eukaryotes [22–24]. Based on their sequence similarity, these proteins have been divided into eight subfamilies, designated A to H [19,23,25]. ABC transporters can not only translocate a wide variety of substrates but also take on other roles, including cell signaling and ribosome assembly and translation [24,26]. Moreover, the subfamilies B, C, and G contain pumps that are capable of mediating drug transport [19,23,26–28]. Considerable evidence has shown that the absorption, distribution and elimination of MLs in hosts and parasites are under the control of multidrug resistance (MDR) transporters, a group of ABC transporters that includes P-glycoproteins (Pgps) [29].

P-glycoprotein (Pgp), encoded by *MDR1* or *ABCB1*, was first discovered in the ovary cells of colchicine-resistant Chinese hamster in 1976 [30]. It plays a crucial role in protecting tissues from toxic xenobiotics and endogenous metabolites, and also affects the uptake and distribution of many important drugs [31,32]. Broad substrate specificity is a hallmark of Pgp, and naturally occurring abamectin and ivermectin are suitable substrates for Pgp [33–36]. Pgp has been implicated in avermectin resistance in a number of parasites and insects [37–40]. Moreover, overexpression of Pgp has been documented in the resistant strains of several insect species, including the spider mite, *Tetranychus urticae*, the salmon louse, *Lepeophtheirus salmonis*, the tobacco budworm, *Heliothis virescens*, and the cotton bollworm, *Helicoverpa armigera*, which is consistent with the drug-resistant nematodes [28,37–39,41–45]. Most recently, Luo (2013) [46] suggested that elevated expression of Pgp plays a crucial role in abamectin resistance in *Drosophila*. To study the function of Pgp in *P. xylostella*, we cloned a cDNA encoding a Pgp in *P. xylostella*, quantified the mRNA expression profiles in different tissues and developmental stages and investigated the transcriptional response of *PxPgp1* after exposure to abamectin.

## 2. Results and Discussion

### 2.1. Molecular Cloning of *PxPgp1*

The full-length cDNA sequence of a Pgp gene from *P. xylostella* was obtained and named *PxPgp1*. It has a 3774 bp open reading frame (ORF), a 133 bp 5′-untranslated region (UTR) containing a TATA box, and a 258 bp 3′-UTR containing a 32 bp poly-A tail. A classic polyadenylation signal, AATAAA [47], is located 18 bp upstream of the poly-A tail. The *PxPgp1* cDNA encodes a 1257-amino acid peptide with a molecular weight of 137.775 kDa and an isoelectric point of 5.71. The deduced protein has two distinct sections, which mirror each other and is comprised of a transmembrane domain (TMD) containing multiple transmembrane regions and a nucleotide-binding domain (NBD). The TMDs and NBDs were arranged in the *N*- to *C*-terminus order of TMD-NBD-TMD-NBD, which is the classical domain architecture of a full transporter. By coupling and hydrolyzing ATP, NBDs provide energy and work together with TMDs to remove excessive substrates. Generally, two molecules of ATP are consumed during a single transportation cycle [48,49].

**Figure 1:**
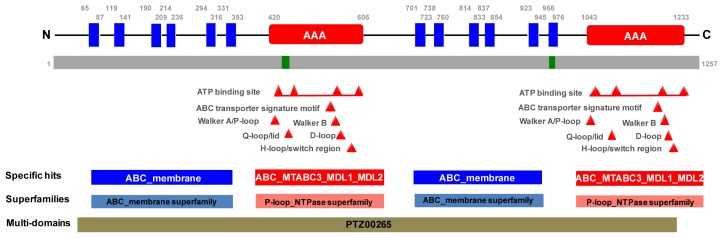
Schematic drawing of the primary and secondary structures of *PxPgp1*. TMpred and the conserved domain database from NCBI were used to construct this map. Signature motifs of the ABC superfamily are color-coded, including transmembrane domains (blue), a nucleotide domain (red) and regions with low complexity (green). PTZ00265 in grey denotes a multidrug-resistance protein (mdr1).

Based on a preferred model from TMpred, *N*-terminus inside, the primary structure of *PxPgp1* contains 12 transmembrane helices. However, it did not have a signal peptide at the *N*-terminal (Figure 1). The secondary structure has two sections, each one includes an ABC signature motif, a Walker A motif, a Walker B motif, a D-loop, a Q-loop and an H-loop (Figure 1) [50]. Although the tertiary structure of *PxPgp1* could not be fully resolved due to a lack of an optimal peptide template (Figure S2), the majority of the transmembrane regions and the second NBD were simulated based on the crystal structure of a multidrug transporter P-glycoprotein, 3g5u, from *Caenorhabditis elegans* [33,51]. The two unmatched structural features, the sixth transmembrane domain (TM6) and the first NBD (NBD1) might be unique to *P. xylostella* (Figure S2). Sheps *et al.* [27] suggested that full-transporters generally evolved from half-transporters, whereas half-transporters evolved from duplicated genes; the ABCB subfamily contains both full- and half-transporters. It was also hypothesized that duplications of ABCB genes gave rise to the Pgp genes [27]. Although the full-length sequences of *PxPgp1* in the resistant and susceptible strains differed at some nucleotide sites, no consistent differences were identified [27].

### 2.2. Phylogenetic Relationship of *PxPgp1* with Other Insect Pgps

**Figure 2:**
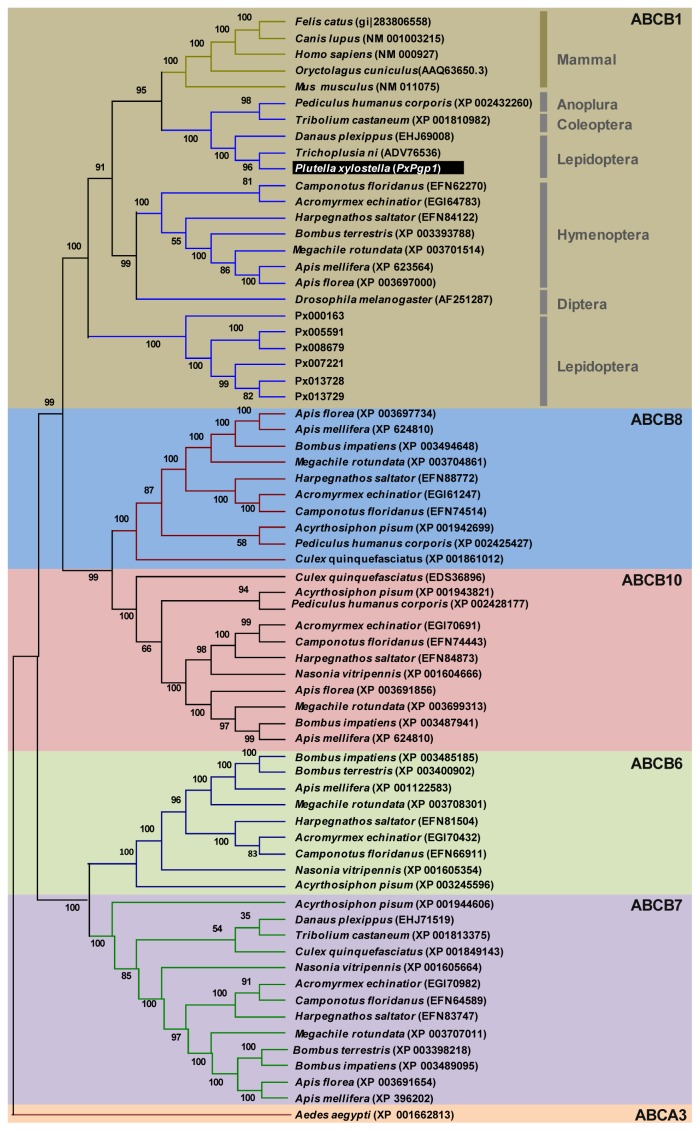
Phylogenetic relationship of *PxPgp1* with other insect Pgps. The percentage of replicate trees in which the associated taxa clustered together in the bootstrap test (1000 replicates) is shown next to the branches [52]. Evolutionary analyses were conducted in MEGA5.05 [53].

Phylogenetic analysis clustered *PxPgp1* with *ABCB1* genes from *P. xylostella* genome, other insect species and mammal Pgps from the ABCB subfamily, within the ABCB1 subgroup (Figure 2). Among them, *PxPgp1* had the highest sequence similarity with two other lepidopteran species, the cabbage looper, *T. ni* (74%) and the monarch butterfly, *D. plexippus* (71%), which are substantially higher than its similarity with Pgps from other insect orders, including 10 hymenopteran insects (44%–48%), the human body louse, *Pediculus humanus corporis* (49%) and the red flour beetle, *T. castaneum* (51%).

### 2.3. Expression Profiling of *PxPgp1* in Different Developmental Stages and Tissues

**Figure 3:**
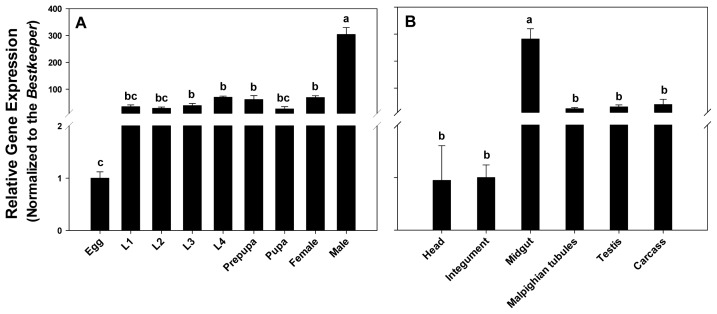
Expression profiles of *PxPgp1* in different developmental stages and tissues. Distribution of *PxPgp1* in different developmental stages of *P. xylostella* is depicted in (**A**). The mRNA quantity is expressed relative to the egg stage. L1 to L4 denote the first- to fourth-instar larvae, respectively. The relative expression levels of Pgp in various tissues of fourth-instar larvae are shown in (**B**). Data are presented as mean ± SE for three independent replicates. Letters denote levels of statistical significance, according to Tukey’s test (*p* < 0.05).

*PxPgp1* was constantly expressed during the entire life cycle of *P. xylostella* and the expression level increased continually during the larval stage, which was consistent with the expression profile in *Heliothis virescens* [42]. Similar to the expression pattern of an orthologue in the salmon louse *L. salmonis* [41], *PxPgp1* in adult males were significantly higher than that in other developmental stages (ANOVA and Tukey’s, *p* < 0.05), which were 7.82, 4.33, 4.94 and 4.35 times higher than that in the third-instar larvae, fourth-instar larvae, prepupae and adult females, respectively (Figure 3A).

The relative expression level of *PxPgp1* were highest in the midgut (*p* < 0.05), which was about 11.83-, 9.37-, and 7.24-fold higher than that in the malpighian tubules, testes and carcass, respectively (Figure 3B). *PxPgp1* expressions in the head and integument were equally low. This is consistent with the other lepidopteran, the cabbage looper, *T. ni*, in which Pgp was most abundant in the midgut and followed by the malpighian tubules [54]. Pgp was also highly expressed in the malpighian tubules of *Manduca sexta* larvae [55]. Two Pgp genes in *D. melanogaster*, *mdr49* and *mdr65*, were located in the brain and gut tissues, respectively [56]. Spatial distribution of these multidrug resistant proteins limits xenobiotic absorption and decreases the penetration of xenobiotics from the systemic circulation, thus protecting vital structures such as the brain or testes against toxins [57,58].

### 2.4. Transcriptional Response of *PxPgp1* to Abamectin Exposure

#### 2.4.1. Acute Response of *PxPgp1* to Abamectin Treatment

**Figure 4:**
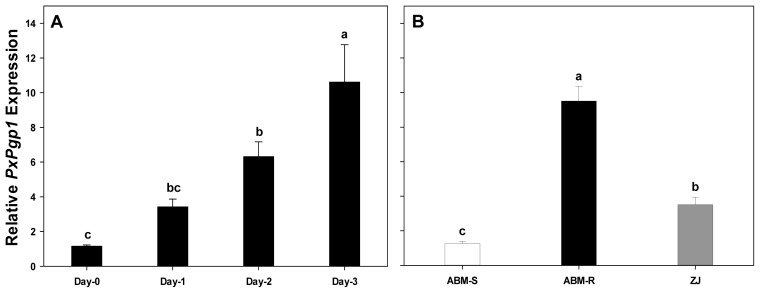
Transcriptional response of *PxPgp1* to abamectin. Relative gene expressions of *PxPgp1* after treatment with abamectin are shown in (**A**). The constitutive expressions of *PxPgp1* in abamectin-resistant and -susceptible *Plutella xylostella* is shown in (**B**). ABM-R and ABM-S represent laboratory-selected abamectin resistant and susceptible *P. xylostella* strains, respectively, and ZJ is field strain collected from Zhejiang Province, China. Data are presented as means ± SE. Letters denote levels of statistical significance in expression levels according to Tukey’s test (*p* < 0.05).

To investigate the acute transcriptional response of *PxPgp1*, third-instar larvae of *P. xylostella* were treated with abamectin at a concentration of LC_50_ of 25 μg/L (ABM-S, Table 1). The relative expression levels of *PxPgp1* over a 3-day period (Day-1, -2 and -3) were 3.14-, 6.31- and 10.61-fold higher than Day-0 (Figure 4A).

#### 2.4.2. Constitutive Expression of *PxPgp1* in Abamectin-Resistant and -Susceptible *Plutella xylostella*

Two laboratory strains, ABM-R and ABM-S, and one field population, ZJ, were used to investigate the expression profiles of *PxPgp1* in abamectin-resistant and -susceptible *P. xylostella*. In comparison to the susceptible ABM-S strain, the resistance ratio of ABM-R and ZJ were 217.7 and 23.1, respectively (Table 1). *PxPgp1* expression levels in the first filial generation (F1) of ABM-R and ZJ were 9.51- and 3.51-fold higher than that in the susceptible strain, respectively (Figure 4B).

Drug transporters play an important role in drug resistance, usually showing elevated expression levels. In this study, mRNA expression levels in the susceptible strain were increased after exposure to abamectin in a dose (day)-dependent manner. The expression levels of *PxPgp1* in susceptible individuals significantly increased when treated with abamectin at LC_50_. Furthermore, both laboratory-selected and field-collected abamectin-resistant and -susceptible strains showed distinct differences in their expression levels of *PxPgp1. PxPgp1* was consistently expressed at higher levels in the abamectin-resistant strains, with or without exposure to the insecticide. This suggests that elevated expression of *PxPgp1* is not only an instantaneous response of *P. xylostella* to abamectin exposure, but also an existing mechanism to cope with the insecticide challenge in the field. Elevated expression of Pgps in avermectin-resistant strains has been documented in many parasitic nematodes and insects. For example, increased expression of Pgp was found in an ivermectin-resistant strain of *Haemonchus contortus* [59]. James and Davey also reported that ivermectin resistance is associated with elevated expression of Pgps in *Caenorhabditis elegans* [45]. In thiodicarb-resistant tobacco budworm, *H. virescens*, Pgp expression was substantially higher than their susceptible counterpart [42]. The expression of a Pgp (*mdr49*) mRNA can be induced by colchicine, a toxic natural product and secondary metabolite, in *D. melanogaster* [56]. In recent years, Pgp-mediated multidrug resistance has been reversed by RNA interference (RNAi), a functional genomics tool, both *in vivo* and *in vitro* [60–62].

## 3. Experimental Section

### 3.1. Plutella Xylostella Strains

*Plutella xylostella* strains, ABM-S and ABM-R, were originally collected in 1990 from a cabbage (*Brassica* sp.) field in Guangdong Province, China. The susceptible ABM-S strain had never been exposed to abamectin, while the resistant ABM-R strain was selected continuously with insecticide treatment. The larvae of *P. xylostella* were reared on Chinese cabbage leaves at 25 ± 1 °C, a relative humidity (RH) of 60%–70% and a photoperiod of 16:8 h (L:D). Adults were provisioned with a 10% honey solution [15]. ZJ strain was originally collected from Zhejiang Province, China.

### 3.2. Chemicals and Bioassay

Abamectin (containing 93% avermectin B1a and 7% avermectin B1b) was obtained from Department of Applied Chemistry, the China Agricultural University (CAU). Leaf residue bioassays were carried out on glass plates using the third-instar larvae. To establish the dose-response curve, pesticide-free cabbage leaves and different doses of abamectin were applied. Bioassays ran for 3 days, and the cumulative mortality was documented at the endpoint [63].

### 3.3. Molecular Cloning of Pgp

Total RNA was isolated from the fourth-instar larvae of *P. xylostella* using a Trizol kit (Invitrogen, Carlsbad, CA, USA) according to the manufacturer’s instruction. First-strand cDNA was synthesized using a PrimeScript II 1st strand cDNA synthesis kit with oligo dT primers (Takara Biotechnology, Dalian, China). Cloning was carried out in three steps integrating primer walking with the rapid amplification of cDNA ends (RACE) PCR. First, two sets of degenerate primers (primers 1 and 2 in Table 2) were used to generate the two conserved fragments, S1 and S2 (Figure S1). Then, a pair of gene specific primers was designed to amplify the gap (G1). The 3′- and 5′-terminus (fragments I and II) were obtained using a SMARTer^™^ RACE cDNA Amplification kit following the manufacturer’s protocol (Takara Biotechnology, Dalian, China). Finally, to validate the cloning result, a pair of gene specific primers covering the entire full-length cDNA sequence of Pgp was used to amplify a long fragment (PCR III) containing the putative open reading frame (Figure S1). Primer Premier 5.0 (Premier Biosoft International, Palo Alto, CA, USA) were used to design the above mentioned primers (Table 2). Amplified PCR products were cloned into a pEASY^™^-T5 vector (Trans Gene Biotech, Beijing, China) and sequenced by Tsingke (Beijing, China). Full-length cDNAs of ten individuals including five clones from each *P. xylostella* were sequenced.

### 3.4. Bioinformatics Analysis

Sequences alignment was performed with DNAMAN 7.0 (Lynnon Biosoft, Quebec, Canada). The isoelectric point and molecular weight of the deduced protein were estimated using the ExPASy Proteomics Server [64,65]. SignalP 4.1 Server [66] was used to predict the signal peptide. The primary and secondary structures of *PxPgp1* were resolved by TMpred [67] and conserved domains were detected by the NCBI sequence analysis tools [68]. The tertiary structure of *PxPgp1* was simulated by SWISS-MODEL [69,70] using a readily available template from RCSB Protein Data Bank (PDB). PyMOL-v1.3r1 [71] was used to visualize the 3-D structure and to label important structural features. To investigate the phylogenetic relationship of *PxPgp1* with *Pgps* from other insect species, a neighbor-joining tree was constructed [72]. The peptide sequences of ABC-B orthologs were extracted from the GenBank and *P. xylostella* genome [73]. Although 17 transcripts from *P. xylostella* genome were categorized into ABCB1, only 6 genes (*Px007221*, *Px005591*, *Px013728*, *Px013729*, *Px008679*, *Px000163*) containing complete ORF were selected for the subsequent phylogenetic analysis. Amino acid sequences were aligned using MEGA 5.05 [53] and ClustalW [74], and bootstrap values were calculated after 1000 replications [52].

### 3.5. Quantitative Real-Time RT-PCR

Relative expression levels of *PxPgp1* in different developmental stages, in various tissues and in larvae treated with abamectin were examined using quantitative real-time reverse transcription polymerase chain reaction (qRT-PCR). For different developmental stages, eggs, first- to fourth-instar larvae, pre-pupa, pupae and 1-day-old adult female and males were collected. For tissue parts, the head, integument, midgut, malpighian tubules, testes and carcass of fourth-instar larvae were dissected in cold phosphate buffered saline (PBS; 137 mmol/L NaCl, 2.7 mmol/L KCl, 10 mmol/L Na_2_HPO_4_, 2 mmol/L KH_2_PO_4_) and washed three times in PBS. Tissues were sampled from 50 individuals for each biological replicate. All samples were snap frozen in liquid nitrogen before stored at −80 °C for the subsequent total RNA isolation. To investigate transcriptional response of *PxPgp1*, third-instar larvae from a susceptible strain were exposed to the median lethal concentration (LC_50_) of abamectin for 1, 2 or 3 days. Moreover, constitutive expression profiles of *PxPgp1* in the abamectin-resistant and susceptible strains were compared using qRT-PCR analysis.

qRT-PCR was conducted using an ABI PRISM 7500 Real-time PCR System (Applied Biosystems, Foster, CA, USA). According to Fu (2013) [75], elongation factor 1 (*EF1*), ribosomal protein L32 (*RPL32*), ribosomal protein S23 (*RPS23*), ribosomal protein S13 (*RPS13*), and β-actin (*ACTB*) were selected as reference genes (Table 3). Specifically, *EF1*, *RPL32* and *RPS23* were used to normalize transcripts among developmental stages and various tissues. *EF1*, *RPS13* and *RPL32* were suited for evaluation of target gene expression after exposure to abamectin. Finally, relative gene expression levels between abamectin-resistant and susceptible strains were evaluated using *EF1*, *ACTB* and *RPL32* as references. All qRT-PCR analyses were run in triplicate for both technical and biological replicates. The qRT-PCR was carried out in a 25 μL reaction containing 1.0 μL cDNA (200 ng/μL), 12.5 μL 2 × SuperReal PreMix Plus, 0.5 μL 50 × ROX Reference Dye, 0.75 μL forward primer (10 μM), 0.75 μL reverse primer (10 μM) and 9.5 μL RNase-free ddH_2_O, following the instructions of the SuperReal PreMix Plus (SYBR Green) kit (Tiangen, Beijing, China). The thermal cycling conditions were 15 min of polymerase activation at 95 °C, followed by 40 cycles of denaturation at 95 °C for 30 s, annealing at 58 °C for 30 s and elongation at 72 °C for 35 s. The amplification efficiency was estimated using the equation: *E* = [10^(−1/slope)−1] × 100%, where slope was derived from plotting the cycle threshold (*C*_t_) value versus six serially diluted template concentrations. Quantification of transcript levels of the Pgp gene was conducted according to the 2^−ΔΔ^*^Ct^* method [76].

### 3.6. Statistical Analysis

The gene expression data were analyzed with ANOVA, and the means were separated by Tukey’s test for significance (*p* < 0.05) using SPSS 19.0 for Windows (SPSS Inc.: Chicago, IL, USA). The LC_50_ value was calculated using a Probit analysis software [77].

## 4. Conclusions

In this study, the full length cDNA of *PxPgp1*, a P-glycoprotein in *P. xylostella*, a devastating vegetable insect pest worldwide, has been cloned and characterized. As a full ABC transporter, phylogenetic analysis places *PxPgp1* with other insect *Pgps* from the ABCB1 subgroup. Spatial and temporal mRNA expression profiling among different tissues and developmental stages suggest *PxPgp1* is most abundant in midgut and is highly expressed in adult males. Exposure to abamectin, a macrocyclic lactone derivative with potent anthelmintic and insecticidal properties, significantly induced the expression of *PxPgp1* in *P. xylostella*, implicating the involvement of *PxPgp1* in the acute response to insecticide treatment. More importantly, the constitutive overexpression of *PxPgp1* in the abamectin-resistant *P. xylostella* suggests the potential connection of *PxPgp1* and abamectin resistance. However, future research involving RNAi-based functional characterization is warranted to establish a causal link between *PxPgp1* and abamectin resistance.

## Supplementary Information



## Figures and Tables

**Table 1:** Susceptibility of *Plutella xylostella* strains to abamectin treatment.

Strain	LC_50_ (95% FL) (mg/L^−1^)	Slope (±SE)	RR a
ABM-S	0.0025 (0.0015–0.042)	1.651 (±0.218)	1
ABM-R	5.442 (3.487–8.493)	1.882 (±0.252)	217.68
ZJ	0.557 (0.242–0.915)	1.682 (±0.221)	23.08

a RR = LC_50_ (strain)/LC_50_ (ABM-S);

RR, resistance ratio; LC_50_, median lethal concentration; FL, 95% Fiducial limits; SE, standard error.

**Table 2:** Location and sequence of primers used for the molecular cloning of *PxPgp1*.

Primer	Position	Sequence (5′-3′)	Length (bp)
1	1060 1566	F: TAYGCTCTKGCMTTCTGG R: GGYTCCTGACYCACSASRCC	506
2	3289 3722	F: AGYGGCTGYGGVAAGAGYAC R: CTTGVACMACCTTTTCACTTTC	433
3	1429 3309	F: CGGCAAGTCGACCATCATAC R: GTACTCTTCCCGCAGCCGC	1880
GSP1	3645	GCTGAGACGACCGAAGATGCTCCTAC	520
GSP2	1273	TAGCAGGGGGTTGATGGACGGCAC	1273

**Table 3:** Primers used for qRT-PCR.

Gene	Accession number	Primer sequence (5′-3′)
*ACTB*	AB282645	F: TGGGTATGGAATCTTGCGG R: GGACATGACGGTGTTGGCG
*EF1*	EF417849	F: GCCTCCCTACAGCGAATC R: CCTTGAACCAGGGCATCT
*GAPDH*	AJ489521	F: GCCACCACTGCCACTC R: CGGGACGGGAACACG
*RPL32*	AB180441	F: CCAATTTACCGCCCTACC R: TACCCTGTTGTCAATACCTCT
*RPS13*	AY174891	F: TCAGGCTTATTCTCGTCG R: GCTGTGCTGGATTCGTAC
*RPS23*	AB180672	F: ATGGGCTGACAAGGATTAC R: TGCGGATGGCAGAGTT
*PxPgp1*		F: GGAATAGTGGCACAAGATGG R: TCTGGAGTCCTGGAGTTATCG
