# Supplementary material for: Molecular Cloning and Characterization of a P-Glycoprotein from the Diamondback Moth, Plutella xylostella (Lepidoptera: Plutellidae)
Source: Int J Mol Sci. 2013 Nov 20;14(11):22891–905. doi: 10.3390/ijms141122891 (PMC3856097; doi:10.3390/ijms141122891)
Supplement: Supplementary file 1 [file ijms-14-22891-s001.pdf]

Supplementary Information

Figure S1. Cloning strategy for *PxPgp1*. The full-length cDNA of *PxPgp1* was cloned through five rounds of PCR. Fragments S1, S2 and G1 were the PCR products, and fragments I and II were obtained by 5'- and 3'-RACE, respectively. PCR III fragment was amplified with specific primers designed according to the assembled full-length cDNA sequences of *PxPgp1*.

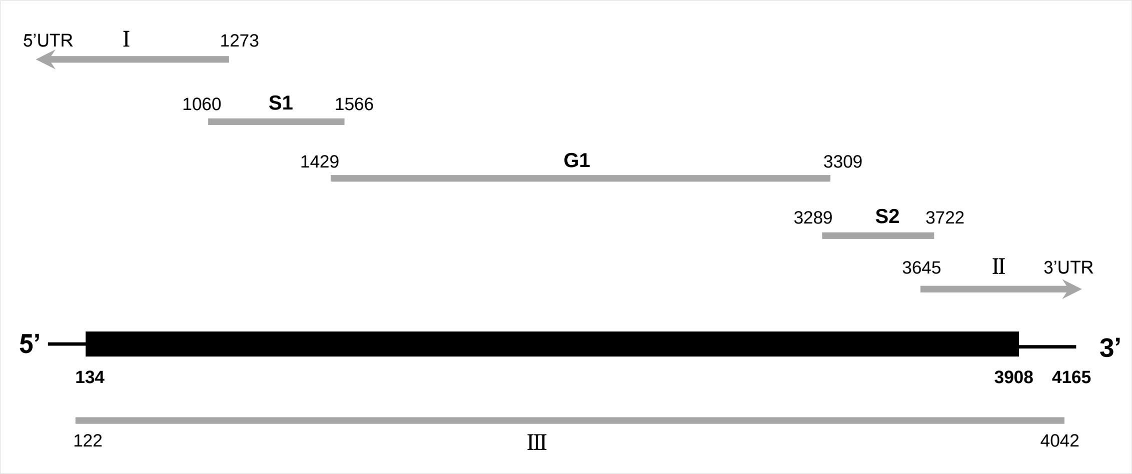

Figure S2. Tertiary structure of *PxPgp1*. This three-dimensional structure was simulated based on an optimal protein template, 3g5u, which was selected from SWISS-MODEL (http://swissmodel.expasy.org/).

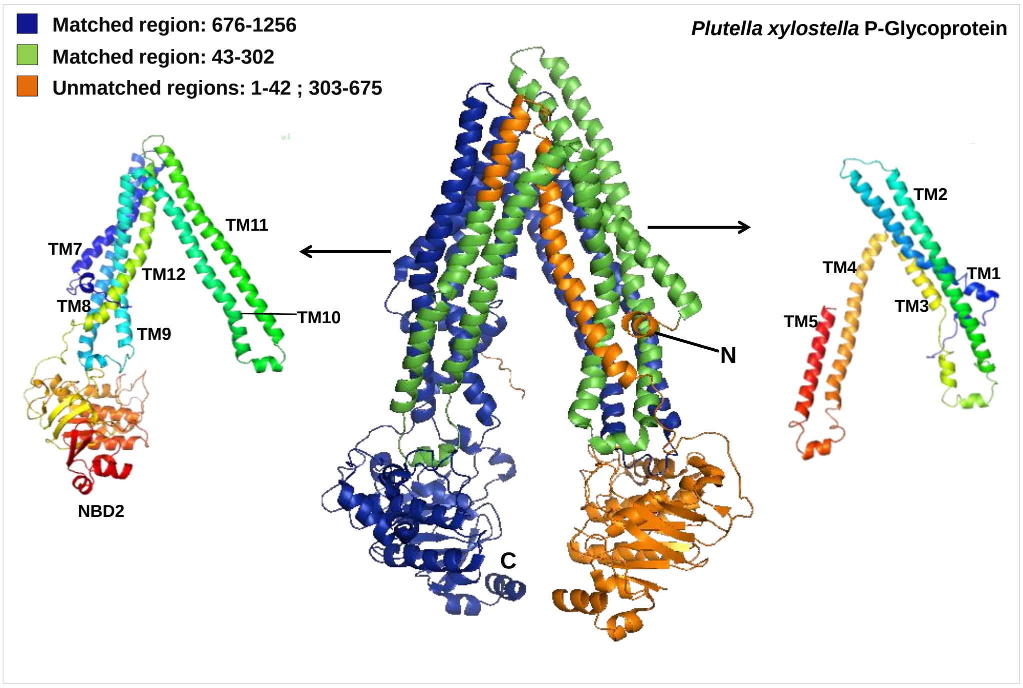

© 2013 by the authors; licensee MDPI, Basel, Switzerland. This article is an open access article distributed under the terms and conditions of the Creative Commons Attribution license (http://creativecommons.org/licenses/by/3.0/).
